# The effect of hypercapnia on the directional sensitivity of dynamic cerebral autoregulation and the influence of age and sex

**DOI:** 10.1177/0271678X231203475

**Published:** 2023-09-25

**Authors:** Ronney B Panerai, Aaron Davies, Rebecca H Clough, Lucy C Beishon, Thompson G Robinson, Jatinder S Minhas

**Affiliations:** 1 Department of Cardiovascular Sciences, 4488 University of Leicester, Robert Kilpatrick Clinical Sciences Building, Leicester, UK; 2 NIHR Leicester Biomedical Research Centre, BHF Cardiovascular Research Centre, Glenfield Hospital, Leicester, UK

**Keywords:** Cerebral blood flow, cerebral autoregulation, hypercapnia, sex, age

## Abstract

The cerebral circulation responds differently to increases in mean arterial pressure (MAP), compared to reductions in MAP. We tested the hypothesis that this directional sensitivity is reduced by hypercapnia. Retrospective analysis of 104 healthy subjects (46 male (44%), age range 19–74 years), with five minute recordings of middle cerebral blood velocity (MCAv, transcranial Doppler), non-invasive MAP (Finometer) and end-tidal CO_2_ (capnography) at rest, during both poikilocapnia and hypercapnia (5% CO_2_ breathing in air) produced MCAv step responses allowing estimation of the classical Autoregulation Index (ARI_ORIG_), and corresponding values for both positive (ARI_+D_) and negative (ARI_−D_) changes in MAP. Hypercapnia led to marked reductions in ARI_ORIG_, ARI_+D_ and ARI_−D_ (p < 0.0001, all cases). Females had a lower value of ARI_ORIG_ compared to males (p = 0.030) at poikilocapnia (4.44 ± 1.74 vs 4.74 ± 1.48) and hypercapnia (2.44 ± 1.93 vs 3.33 ± 1.61). The strength of directional sensitivity (ARI_+D_-ARI_−D_) was not influenced by hypercapnia (p = 0.46), sex (p = 0.76) or age (p = 0.61). During poikilocapnia, ARI_+D_ decreased with age in females (p = 0.027), but not in males. Directional sensitivity was not affected by hypercapnia, suggesting that its origins are more likely to be inherent to the mechanics of vascular smooth muscle than to myogenic pathways.

## Introduction

Cerebral autoregulation (CA), which tends to maintain cerebral blood flow (CBF) relatively constant, despite changes in mean arterial blood pressure (MAP), has been regarded as a protective mechanism that can prevent cerebral ischemia due to reductions in MAP and minimise the risks of capillary damage and cerebral oedema due to arterial hypertension.^1^ The protective aspect of CA has been corroborated by the identification of *directional sensitivity,* as an important additional feature.^2,3^ Accordingly, more efficient control of CBF seems to be obtained for increases in MAP that could have damaging effects on the micro-circulation, as compared to reductions in MAP. Evidence of this behaviour has been reported for both static CA, involving steady-state values of MAP and CBF (or cerebral blood velocity),^4,5^ and dynamic CA, when the MAP-CBF/velocity relationship is described for beat-to-beat measurements.^6–10^ In dynamic CA studies, directional sensitivity has been identified for large excursions in MAP induced by the squat-stand manoeuvre.^7,9,11^ Despite the excellent signal-to-noise ratio afforded by the squat-stand manoeuvre, it also presents limitations due to the difficulty of performing simultaneous physiological interventions^12^ or assessments in patients who cannot comply with its demands. More recently, a novel modelling approach has been proposed to obtain estimates of directional sensitivity of dCA at rest, in measurements with spontaneous fluctuations in MAP and cerebral blood velocity in the middle cerebral artery (MCAv).^8^ This approach is particularly advantageous to facilitate studies of directional sensitivity during physiological interventions that could shed light on the origins and physiological basis of this intriguing phenomenon. Of the many different physiological stimuli that can perturb dCA,^1^ hypercapnia takes pride of place due to its well-known effect on depressing dCA,^13,14^ often being regarded as a surrogate of impaired CA.

Given our limited understanding of the mechanisms underlying directional sensitivity, its response to hypercapnia, in comparison with poikilocapnia, could provide clues to its origins. Since hypercapnia is known to depress dCA,^13,15^ the main rationale of our study is to test if directional sensitivity is also blunted by hypercapnia. If that is the case, the origins of directional sensitivity could be associated with concurrent myogenic or metabolic pathways that also control dCA. On the other hand, if an effect is not detected, then we need to look for alternative explanations for this intriguing phenomenon. In addition to shedding light on the possible origins of directional sensitivity of dCA, the study also has the potential to benefit future clinical applications of directional sensitivity analysis, particularly in patients with ischemic stroke, who often present with impaired dCA.^16,17^ The possibility that alterations in directional sensitivity of dCA can add information to standard methods of dCA assessment in patients with ischemic stroke^18^ is of interest and will be discussed in more detail later.

To have the statistical power needed to perform such study,^8^ we have assembled a large number of recordings from three different studies conducted in our laboratory, with identical measurement protocols, to test the hypothesis that hypercapnia would lead to the loss of directional sensitivity. Following previous reports of the effects of age and sex on directional sensitivity,^2,19,20^ we have also included these two potential co-variates in our analyses.

## Methods

### Ethical approval

Ethical approval was obtained from the University of Leicester Ethics Committee (Refs: jm591-c033; 28664-jm591-ls:cardiovascularsciences; 31663-lb330-ls:cardiovascularsciences). All participants provided written, informed consent. All studies were carried out according to the latest approved protocols, the International Conference on Harmonisation-Good Clinical Practice (ICH-GCP), and in accordance with the Declaration of Helsinki, though the study was not registered in a database.

### Participants

The study was based on the amalgamation of three sets of data from the Leicester Cerebral Haemodynamics Database.^21^ In summary, to be included, participants had to be aged 18 years or older; be a member of staff or student at the University of Leicester; be willing to participate, and able and willing to comply with all the study requirements. Female participants who were pregnant, lactating or planning pregnancy were excluded from the study, as well as participants with a history of cardiovascular, neurological or pulmonary disease.

### Experimental protocol

All study participants attended a dedicated cardiovascular research laboratory, which was controlled at a temperature of 20–24°C and was free from distraction. Participants were asked to refrain from heavy meals, strenuous exercise, smoking, alcohol and caffeine for at least 4 hours prior to attending the laboratory.

Once satisfactory signals had been obtained for all equipment, a 5 min recording was performed with subjects resting in the supine position and breathing normally. A second recording was performed with subjects initially breathing normally for 30 s, and then breathing 5% CO_2_ in air with a face mask for 90 s. Breathing 5% CO_2_ in air is the dominant method seen in the literature for inducing hypercapnia and the use of a face mask is well tolerated by most individuals.^12,14,15,22^ These two distinct recording periods provided the data for analyses corresponding to *poikilocapnia* and *hypercapnia*, respectively. Randomization of both conditions was not adopted to avoid the poikilocapnia being influenced by the washout phase of hypercapnia.^15,22^

### Instrumentation

Beat-to-beat, non-invasive BP measurements were recorded using the Finometer cuff device, attached to the middle finger of the right hand (Finapres® Medical Systems; Amsterdam, The Netherlands). The *PhysioCal* mechanism was switched off during recordings to ensure a continuous BP trace. Brachial BP was measured before the recording using sphygmomanometry (UA 767 BP monitor) to calibrate the Finometer signal. Unilateral insonation of the middle cerebral artery (MCA) through the temporal window on the dominant hemisphere was performed using transcranial Doppler ultrasound (TCD, DWL Doppler Box 10.5.1) with 2 MHz probes, held in place by a bespoke head frame. One study also performed measurements in the non-dominant hemisphere, but these were not considered for consistency with the other two studies. HR was measured using three-lead electrocardiogram. Respiratory rate and EtCO_2_ were monitored using nasal cannulae (Salter Labs, ref 4000) attached to a capnograph (Capnocheck Plus). All signals were simultaneously recorded onto the Physiological Data Acquisition System (PHYSIDAS, Leicester Medical Physics Department), at a sampling rate of 500 samples/s, for subsequent offline analysis.

### Data analysis

Data were edited and analyzed using in-house software written in Fortran. Under visual inspection, narrow spikes (<100 ms) and artefacts in the recordings were manually removed by linear interpolation. The Finometer readings were calibrated using the brachial BP values. The MCAv signal was passed through a median filter and all recordings were filtered in the forward and reverse direction using an eighth-order Butterworth low-pass filter with a 20 Hz cut-off frequency. The beginning and end of each cardiac cycle were marked from the ECG signal, and mean values for MAP, HR and MCAv were calculated for every heartbeat. The end of expiration was detected in the EtCO_2_ signal, linearly interpolated, and re-sampled in synchronism with the cardiac cycle. Beat-to-beat parameters were interpolated with a third-order polynomial and then resampled at 5 Hz to produce signals with a uniform time base.

Vasomotor reactivity to CO_2_ (VMR_CO2_) was calculated by the ratio between the change in mean values of MCAv from poikilocapnia to hypercapnia, divided by the corresponding mean values of EtCO_2_.^22^

To assess the directional sensitivity of dynamic CA at rest, separate estimates of the integrated positive (MAP_+D_) and negative (MAP_−D_) derivatives of MAP are needed, as described in detail previously.^8^ In short, the numerical derivatives of MAP are calculated using a 5-sample moving window, then separated into their positive and negative values. The positive and negative derivatives are integrated separately and the linear trends were removed by linear regression, generating the MAP_+D_ and MAP_−D_ signals that are then taken as the two separate inputs to the MCAv output.

**Figure 1.**
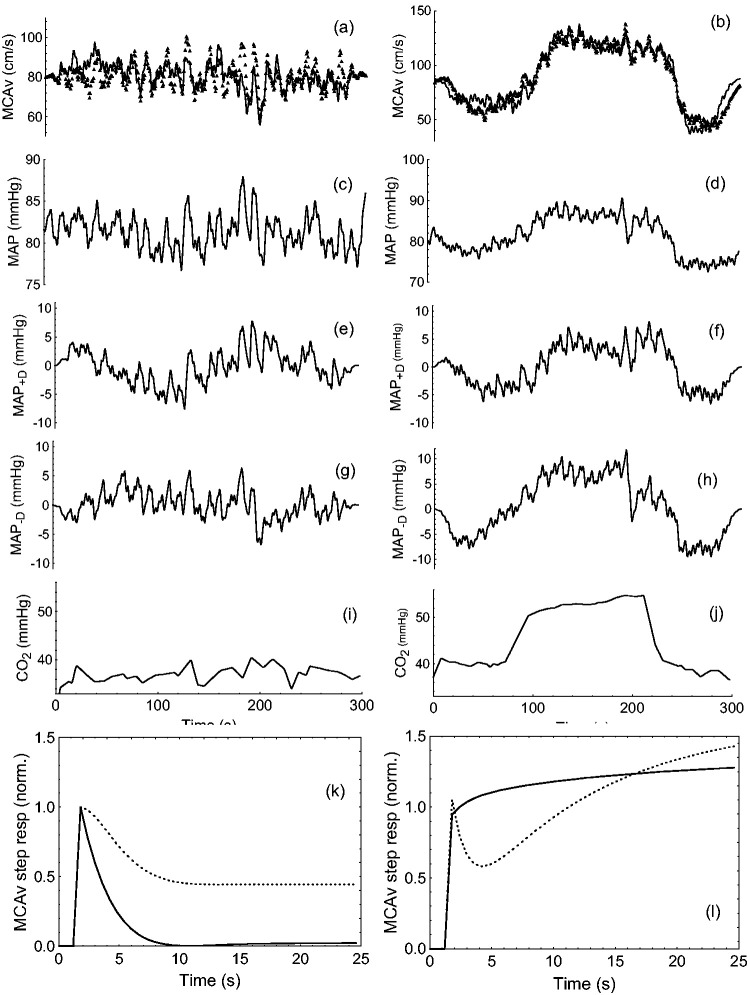
Representative recordings and MCAv step responses from a 52 year old female subject during poikilocapnia (left column plots) and hypercapnia (right column plots). (a,b) middle cerebral artery velocity (MCAv, continuous line) and model predicted output (symbols); (c,d) mean arterial blood pressure (MAP); (e,f) integrated positive derivative of MAP; (g,h) integrated negative derivative of MAP; (i,j) End-tidal CO_2_; (k,l) normalised MCAv step responses for MAP_+D_ (continuous line) and MAP_-D_ (dashed line). The poikilocapnia and hypercapnia, model fittings had R_mod_ of 0.363 and 0.934, respectively. Corresponding values of ARI_+D_/ARI_-D_ were 4.46/3.27 and 0.00/0.64, whilst the NMSE were 0.034/0.024 and 0.093/0.159, respectively.

The temporal relationship between MAP (or the integrated derivatives MAP_+D_ and MAP_−D_) and MCAv was modelled in the time-domain with an autoregressive-moving average (ARMA) structure, as described previously.^8^ In summary, at each point in time, MCAv is expressed by a combination of past values of MCAv (AR terms) and current and past values of the input(s) (MA terms). Based on previous studies, the order of the AR terms was set to two past samples, and the order of the MAP (or the integrated derivatives MAP_+D_ and MAP_−D_) input(s) (MA terms) were set to three terms, corresponding to the present sample and two past samples. This structure was shown to match the second order differential equation model of Tiecks et al.^23–25^ which was adopted to provide the Autoregulation Index (ARI). After model coefficients were estimated by singular value decomposition, they were used to obtain the MCAv response to a step change in MAP, henceforth referred to as the MCAv step response (SRMCAv-MAP). SRMCAv-MAP was obtained for the original MAP signal (SRMCAv-MAP_ORIG_) and also for the integrated positive (SRMCAv-MAP_+D_) and negative (SRMCAv-MAP_-D_) time derivatives of MAP. Therefore, SRMCAv-MAP_+D_ represents the CBFv step response to increases in MAP, whilst SRMCAv-MAP_−D_ corresponds to the step response to reductions in MAP (Figure 1).

For each of these three different types of step responses, the ARI was extracted by fitting the estimated responses to the model templates proposed by Tiecks et al.^23^ yielding corresponding values ARI_ORIG_, ARI_+D_, ARI_−D_ for each of the input variables. The ARI ranges from zero (absence of autoregulation) to nine (best autoregulation observed) and it has been widely used in physiological and clinical studies to express the efficiency of dynamic CA.^8,9,26,27^

### Statistical analysis

Given the limitations of the Shapiro-Wilk W statistic to determine normality of data for large sample sizes, this was ascertained using Q-plots. A bootstrap analysis was performed to establish the 95% confidence limits for the correlation coefficient between the predicted model output and measured MCAv values (R_mod_), as well as for the normalised mean square error (NMSE) between MCAv step responses and the Tiecks model fitting estimation of ARI.^28^ To test the null hypothesis of lack of association between input(s) and output (MCAv), surrogate data were generated by combining the inter-subject signals of MAP_+D_ and MAP_-D_ from one subject with the MCAv signal of different subjects.^29^ Using a subset of 100 paired recordings, this process generated 9900 estimates of R_mod_ and NMSE that were then used to obtain the corresponding 95% confidence limits. For R_mod_ the limit was 0.223. For the NMSE, the joint distribution for MAP_+D_ and MAP_-D_ was taken into consideration by obtaining the 2-D histogram that was smoothed and integrated to obtain the value of NMSE < 0.491 for acceptance of MCAv step responses at the 95% confidence limit.

The effects of different breathing conditions (poikilocapnia or hypercapnia) on ARI_ORIG_, ARI_+D_, ARI_-D_ and other physiological parameters were tested with the General Linear Model (GLM) with sex as categorial and age as continuous predictors using *STATISTICA* (Version 8.0 *StatSoft Inc*. Tulsa, OK, USA) software. Statistical significance was assumed for p < 0.05.

## Results

The three studies extracted from the Leicester Cerebral Haemodynamics database yielded a total of 144 healthy subjects. They all provided recordings suitable for analysis, but 40 subjects had to be removed due to the constraints imposed by the 95% confidence limits for R_mod_ and NMSE, as described above. The reasons for this relatively high attrition rate will be discussed below. The 104 subjects accepted for further analyses ranged in age from 19 to 74 years, without a significant difference in age distribution between the 46 males and 58 females (Table 1). Breathing 5% CO_2_ induced significant hypercapnia, as reflected by the values of EtCO_2_, with corresponding increases in MCAv and heart rate (Table 1). Heart rate and MCAv were higher in females, who also showed an interaction effect of sex with the EtCO_2_ change from poikilocapnia to hypercapnia (Table 1). VMR_CO2_ was higher in females when compared to males (Table 1) and was not influenced by age (p = 0.35).

**Table 1.** Demographic and physiological characteristics for poikilocapnia and hypercapnia.

	Poikilocapnia		Hypercapnia		p-value CO_2_	p-value sex	p-value inter
Variable	Male (n = 46)	Female (n = 58)	Male (n = 46)	Female (n = 58)			
Age (years)	37.8 ± 17.3	36.8 ± 16.1	–	–	–	0.76	–
EtCO_2_ (mmHg)	37.81 ± 4.17	36.14 ± 2.93	41.28 ± 4.37	40.40 ± 3.02	**<0.0001**	0.063	0.090
MCAv (cm.s^−1^)	56.6 ± 13.9	67.8 ± 17.0	61.5 ± 15.8	76.7 ± 20.6	**<0.0001**	**<0.0002**	**0.0035**
MAP (mmHg)	88.6 ± 14.4	89.4 ± 13.0	89.9 ± 12.0	89.3 ± 14.7	0.57	0.99	0.51
HR (bpm)	65.4 ± 12.2	70.1 ± 9.2	65.6 ± 11.1	71.4 ± 10.1	**0.014**	**0.013**	0.093
VMR_CO2_(cm.s^−1^.mmHg^−1^)	–	–	1.68 ± 1.20	2.37 ± 1.37	**–**	**0.014**	–

Values are mean ± SD. EtCO_2_: end-tidal CO_2_; MCAv: middle cerebral artery blood velocity; MAP: mean arterial blood pressure; HR: heart rate; VMR_CO2_: vasomotor reactivity to CO_2_.

p-value CO_2_: effect of hypercapnia; p-value sex; effect of sex, p-value inter: interaction effects of hypercapnia and sex.

P-values <0.05 are highlighted in bold.

**Figure 2.**
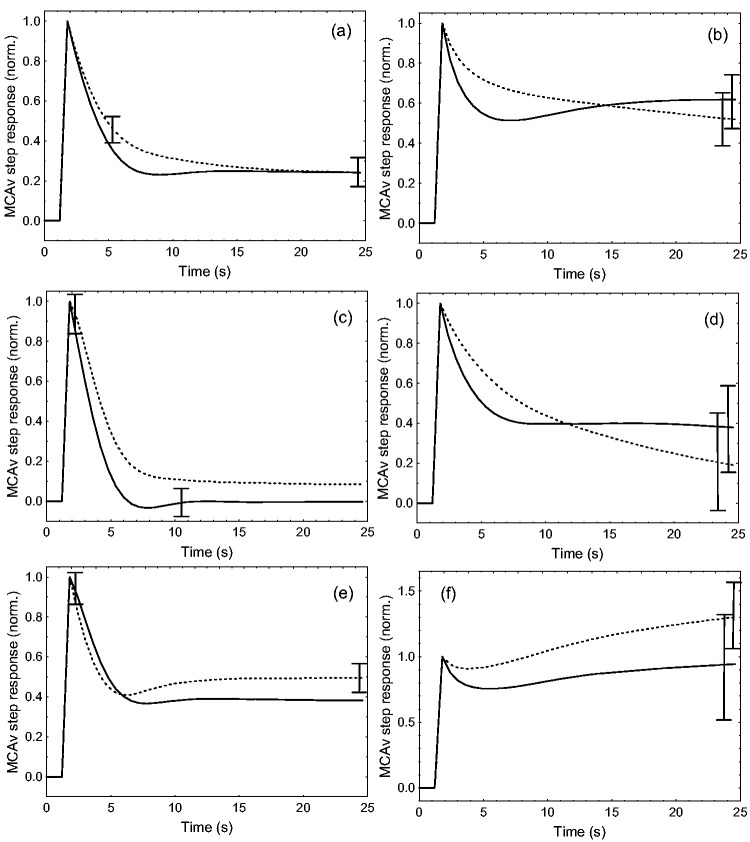
Population average MCAv step responses during poikilocapnia (a,c,e) and hypercapnia (b,d,f) for male (continuous line) and female (dashed line) subjects. (a,b) classical step responses for the mean arterial blood pressure (MAP) input; (c,d) step responses for the integrated positive derivative of MAP; (e,f) step responses for the integrated negative derivative of MAP. The error bars correspond to the largest ± 1 SE at the point of occurrence. Note the different amplitude scale for (f).

ARMA modelling of MCAv for the separate MAP_+D_ and MAP_-D_ inputs had mean ± SD predictive model correlations R_mod_ = 0.375 ± 0.175 (poikilocapnia) and R_mod_ = 0.483 ± 0.228 (hypercapnia) with corresponding values of NMSE for the MAP_+D_ and MAP_-D_ inputs of 0.102 ± 0.086 and 0.134 ± 0.131 (poikilocapnia) and 0.139 ± 0.141 and 0.154 ± 0.152 (hypercapnia), respectively. Directional sensitivity of dynamic CA was identified by MCAv step responses to the MAP_+D_ input suggesting more efficient autoregulation when compared to the MAP_-D_ input (Figures 1 and 2). Noteworthy, this difference between the SRMCAv-MAP_+D_ and SRMCAv-MAP_-D_ showed considerable individual variability as illustrated in Figures 1 and 2 during hypercapnia.

MCAv step responses to the original, single input MAP signal, as well as to the separate MAP_+D_ and MAP_-D_ inputs, showed the expected worsening of dynamic CA due to hypercapnia (Figure 2 and Table 2). Although population averages of SRMCAv suggested a less efficient dynamic CA in females (Figure 2), this was only significant for the original MAP input, as indicated by the values of ARI_ORIG_ (Table 2). Of considerable relevance, directional sensitivity, as reflected by the difference between ARI_+D_ and ARI_-D_, did not show any differences due to hypercapnia or sex (Table 2).

**Table 2.** Autoregulation index (ARI) estimates for poikilocapnia and hypercapnia.

	Poikilocapnia		Hypercapnia		p-value CO_2_	p-value sex	p-value inter
Variable	Male (n = 46)	Female (n = 53^a^)	Male (n = 46)	Female (n = 53^a^)			
ARI_ORIG_	4.74 ± 1.48	4.44 ± 1.74	3.33 ± 1.61	2.44 ± 1.93	**<0.0001**	**0.030**	0.17
ARI_+D_	5.55 ± 1.74	4.82 ± 1.64	3.82 ± 2.38	3.78 ± 2.73	**<0.0001**	0.17	0.30
ARI_-D_	4.29 ± 2.24	3.42 ± 2.39	1.94 ± 2.03	2.26 ± 2.77	**<0.0001**	0.35	0.12
ARI_DIFF_	1.26 ± 2.60	1.40 ± 2.28	1.88 ± 3.15	1.52 ± 3.93	0.46	0.76	0.61

Values are mean ± SD. ARI_ORIG_: original autoregulation index (ARI) for the MAP-MCAv relationship; ARI_+D_: ARI value for the integrated positive derivative of MAP; ARI_-D_: ARI value for the integrated negative derivative of MAP, ΔARI_DIFF_: difference ARI_+D_-ARI_-D_.

p-value CO_2_: effect of hypercapnia; p-value sex: effect of sex, p-value inter: interaction effects of hypercapnia and sex.

a n = 5 ouliers of ARI_DIFF_ = −9 were removed from this analysis. P-values <0.05 highlighted in bold.

**Figure 3.**
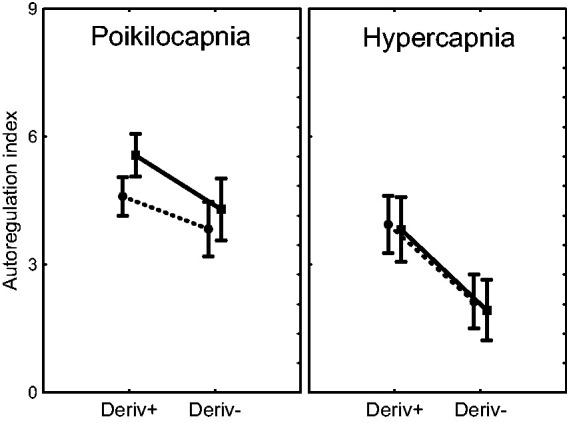
Difference of autoregulation index between the integrated positive (Deriv+) and negative (Deriv−) mean arterial blood pressure (MAP) inputs for the ARMA model of the middle cerebral artery velocity response during spontaneous fluctuations in BP for male (n = 46, continuous line) and female (n = 58, dashed line) subjects. The error bars correspond to the 95% confidence limits.

**Figure 4.**
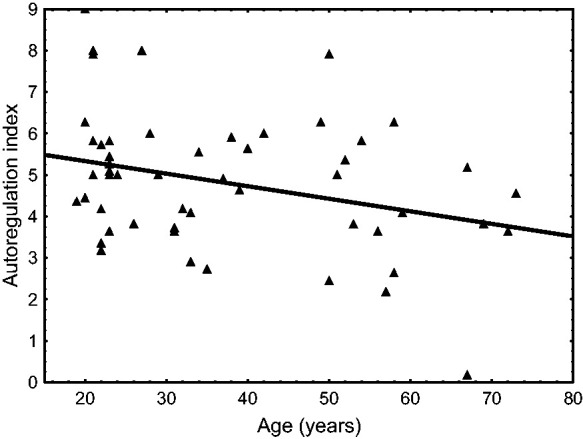
Autoregulation index as a function of age during poikilocapnia in female subjects (n = 53). The symbols and continuous regression line (p = 0.027) correspond to estimates from the integrated positive derivative input to the ARMA model of cerebral blood velocity in the middle cerebral artery. The dashed regression line (p = 0.060) correspond to estimates from the integrated negative derivative input. Corresponding linear regressions for male subjects had p-values of 0.21 and 0.93, respectively.

GLM analysis of ARI_+D_ and ARI_-D_ as dependent variables, indicated highly significant effects of directional sensitivity (p < 0.0001) and hypercapnia (p < 0.0001), without predictive contributions of age or sex. As suggested by Figure 3 though, sex could have had an influence during poikilocapnia, but not during hypercapnia. This possibility was confirmed by performing the GLM during poikilocapnia only, showing significant effects of directional sensitivity (p = 0.011), sex (p = 0.027) and age (p = 0.013). Linear regression analysis demonstrated that the effect of age was restricted to female subjects only (Figure 4).

In summary, our results confirmed that directional sensitivity is present in estimates of dynamic CA at rest, but it is not influenced by hypercapnia, age or sex. We have also found that in females at rest, ARI_+D_ was influenced by age (p = 0.027, Figure 4), but ARI_-D_ was not (p = 0.060).

## Discussion

### Main findings

The use of an ARMA model, to obtain the MCAv response to step changes in MAP, or its integrated derivatives, provided temporal patterns that were physiologically plausible and that suggested a more efficient dynamic CA for increases in MAP, that is, the MAP_+D_ input, when compared to the response to the MAP_-D_ input. These results confirmed the feasibility of identifying the directional sensitivity of dynamic CA in healthy subjects at rest, for the first time in the supine position, since it had been previously reported for the sitting and standing postures only.^8^ Contrary to our main hypothesis though, the strength of directional sensitivity, expressed by the difference ARI_+D_–ARI_−D_ was not influenced by hypercapnia. As discussed in more detail below, this finding has considerable relevance for advancing our understanding of the directional sensitivity phenomenon, mainly regarding the potential pathways involved and the loci of its origin. The study has also added to a growing literature on the role of age and sex on the cerebral circulation with findings that shed light on some contentious aspects that have been reported by others.

### Methodological considerations

Before a more in depth interpretation of our main results, some methodological aspects require consideration as this can set cautionary boundaries to how much extrapolation and speculation would be acceptable.

ARMA modelling of the MAP-MCAv relationship is a well established time-domain method to provide markers of dynamic CA, and have been adopted by a relatively large number of studies.^30^ One advantage of this approach, when compared to alternatives such as transfer function analysis,^31^ is the possibility of including multiple inputs, such as sensorimotor paradigms that can quantify the interaction of neurovascular coupling with dynamic CA.^32^ In the present study, this property of ARMA models was used to quantify the separate contributions of MAP_+D_ and MAP_-D_, thus providing estimates of directional sensitivity at rest.

The use of ARMA models though, is not without its drawbacks.^30^ First of all, there is the matter of model order identification. As in previous applications of the ARMA approach, we have adopted model orders that matched the Tiecks model structure,^23–25^ to make this consistent with the use of ARI as a marker of dynamic CA, as well as the use of the NMSE for acceptance of SRMCAv temporal patterns. Nevertheless, whether alternative choices, or indeed, individualized model orders, could have led to different results, or a smaller number of rejected subjects, is one aspect that warrants further investigation. Secondly, statistical reliability also needs to be taken into consideration. Similarly to transfer function analysis, where the coherence function is used to confirm the validity of estimates of gain and phase,^31^ the validity of an ARMA model needs to be assured by the quality of its output predictions, in this case the extent to which inputs (MAP_+D_ and MAP_-D_) can model the MCAv output. For this purpose, we have used the model predictive correlation coefficient (R_mod_), coupled with the quality of fitting to the Tiecks *et al.* templates, expressed by the NMSE.^23,28^ The use of these figures of merit led to the rejection of 40 subjects. Although the number of rejected subjects might look high, compared to the original cohort (n = 144), it is important to take into consideration that it involved the combined conditions of R_mod_, NMSE (one for each input), and simultaneous acceptance at both poikilocapnia and hypercapnia. Each of these conditions might have been responsible for the elimination of five to seven subjects only, but when combined, the overall number grew to 40. Despite this reduction in sample size, the remaining number of 46 male and 58 female subjects was above the minimum sample sizes that we had previously estimated to detect the presence of directional sensitivity, in the worst case of n = 38, for participants at rest in the sitting position,^8^ and led to highly significant differences between ARI_+D_ and ARI_-D_ (Table 2). Nevertheless, future studies are needed to improve the reliability of estimates of directional sensitivity with the ARMA or alternative approaches, to minimize the rate of rejections, mainly when aiming to perform clinical studies of different cerebrovascular conditions.

### Physiological interpretation

In previous studies, investigators have attributed the phenomenon of directional sensitivity to the sympathetic activation that accompanies rises in MAP, that are not observed when MAP falls.^2,3,7,11,19,33^ Due to the observation that the ‘strength’ of directional sensitivity, expressed by ARI_DIFF_, did not change between sitting, standing and SSM maneuvers,^8^ which should be accompanied by differences in sympathetic drive, we have proposed that directional sensitivity is an inherent characteristic of vascular smooth muscle mechanics, with contraction being much faster than relaxation.^8,34,35^ Nevertheless, it should be kept in mind that the extent of sympathetic activation, and its temporal patterns in different physiological conditions, such as rest and SSM, are not known and further attempts to quantify it, as a determinant of directional sensitivity would be highly desirable. Hypercapnia induces vasodilation, due to vascular smooth muscle relaxation, independently of endothelial integrity^36^ but it does not reduce the contractility of vascular smooth muscle cells.^37,38^ Therefore, despite the vasodilation induced by hypercapnia, leading to a depression in dCA, the relative differences between the speeds of contraction and relaxation are maintained, thus explaining the lack of a difference between ARI_+D_ and ARI_-D_ for hypercapnia in comparison with poikilocapnia. Moreover, hypercapnia enhances the vasoconstriction of cerebral arterioles due to sympathetic activity,^39^ and if directional sensitivity was caused by the latter, it could be expected that it would also be enhanced by hypercapnia, which was not the case.

Although hypercapnia often has been used as a surrogate of impaired dCA,^1,13^ its lack of effect on the strength of directional sensitivity cannot be extrapolated to other physio-pathological conditions where there could be disruption of calcium pathways or impairment of pathways governing vascular smooth muscle mechanics. Further studies are needed in patient groups with cerebrovascular conditions, notably moderate/severe ischemic stroke, to improve our understanding of the origins of directional sensitivity.

### Influence of sex on the directional sensitivity of autoregulation

The observation that females had a higher MCAv, in comparison with males (Table 1), has been consistently reported in the literature in studies with moderate (n > 25) to large (n > 200) sample sizes.^40–45^ On the other hand, reports on the influence of biological sex on dCA and CO_2_ reactivity, have been much less consistent and their diversity and specificity, to age and other factors, would make for a much more extensive discussion than is warranted in the context of this study. To focus on directional sensitivity, we are only aware of the study of Labrecque *et al.* which enrolled 10 men and 8 women and did not find a difference due to sex based on the ΔMCAv_T_/ΔMAP_T_ marker of directional sensitivity, although the authors acknowledged the possibility of a type-II error due to the small sample size.^19^ Using a larger sample, we have detected the effect of sex on directional sensitivity during poikilocapnia (Figure 3), but not during hypercapnia. For both conditions, ARI_ORIG_ was lower in females but there were no differences in comparison with males for ARI_+D_, ARI_-D_ or ARI_DIFF_ (Table 2). Curiously, VMR_CO2_ was higher in females (Table 1), highlighting the fact that CO_2_ reactivity and dCA express different mechanisms. Nevertheless, the greater sensitivity to the CO_2_ rise due to 5% CO_2_ breathing in females and the corresponding greater vasodilation that it would generate, could explain why dCA was worse in females during hypercapnia (ARI_ORIG_ in Table 2). These findings raise further questions about the mechanism(s) whereby sex can influence cerebral hemodynamics and, in particular, the directional sensitivity of dCA at rest.

### Influence of age on directional sensitivity of autoregulation

The literature on the influence of age on cerebral hemodynamics has similarities to that of sex, regarding the consistency of reports of the reduction of MCAv with age and the lack of consistency on the effects of age on dCA. In our sample, MCAv and ARI_ORIG_ were not influenced by age, either during poikilocapnia or hypercapnia, even when controlled for sex. Our findings agree with the dominant view in the literature, that dCA is not influenced by age.^46–48^ In a previous study, the ARI_ORIG_ did not show differences due to sex, but in 104 males there was a small effect of age, with a linear regression of ARI = 7.05–0.0251xAGE.^49^ When it comes to directional sensitivity, Brassard *et al.*^2^ reported that age was positively correlated with the %ΔMCAvmean/%ΔMAP index during increases in MAP in 58 men performing SSM at 0.05 Hz, but not at 0.10 Hz. However, we are not aware of any other studies where age was associated with a directional sensitivity response. In the global GLM model, age was not a significant determinant of ARI_+D_ or ARI_-D_ (Table 2) but when only poikilocapnia was considered, age had a significant effect (p = 0.027) on the reduction of ARI_+D_ in female subjects, without a similar effect on males (Figure 4). Above all, age did not affect the strength of directional sensitivity, as expressed by ARI_DIFF_ (Table 2).

Our results for the combined influences of age and sex on directional sensitivity of dCA are complex and warrant further investigation. Despite our moderate sample size (n = 104), it is likely that much larger samples will be required to allow modelling for other co-variates of dCA and directional sensitivity, such as PaCO_2_,^43^ hormonal cycle^50^ and fitness level^20^ to be able to explain the diversity of results in the literature.

### Clinical perspectives

The only clinical study of directional sensitivity of CBF regulation, that we are aware of, concluded that directional sensitivity was present in patients suffering head injury, but not in healthy subjects.^6^ Given that several more recent studies have demonstrated the occurrence of directional sensitivity in healthy subjects, and the fact that they quantified directional sensitivity from the gain of critical closing pressure changes,^6^ this sets the Aaslid study apart from the mainstream literature in this area. The dearth of clinical studies could be partially explained by the difficulty in patients complying with the SSM protocol. However, the possibility of deriving estimates of ARI_+D_ and ARI_-D_ from spontaneous fluctuations in BP and MCAv at rest,^8^ paves the way for expanding the assessment of dCA by adding directional sensitivity information in a myriad of clinical conditions. The main drive to justify such studies would be the possibility that ARI_+D_ and ARI_-D_ would provide additional information to the classical ARI_ORIG_. These could be manifested by changes in the strength of directional sensitivity, as expressed by the difference ARI_+D_ - ARI_-D_, or by one of these metrics responding differently from the ARI_ORIG_ for different maneuvers or patho-physiological conditions. One important example is the management of acute ischemic stroke when complex patho-physiological conditions make it difficult to have a ‘one-size-fits-all’ approach for setting MAP targets.^1^ Although somewhat speculative at this stage, the dilemma about whether to increase MAP to aid perfusion, or reduce it, to prevent secondary damage, is an example of how directional sensitivity information might contribute to more focused future research. As shown previously,^8^ and also in this study (Table 2), ARI_ORIG_ tends to stay between the values of ARI_+D_ and ARI_-D_. Therefore, when ARI_ORIG_ is found to be reduced in acute ischemic stroke, the way corresponding changes in ARI_+D_ and ARI_-D_ are taking place could provide clues to support decision-making. If ARI_+D_ remains approximately constant, and the main drop is observed in ARI_-D_, one would be more concerned with maintaining a relatively elevated MAP, to avoid hypoperfusion due to falls in BP, and vice-versa, if the fall in ARI_ORIG_ resulted mainly from a substantial reduction in ARI_+D_. Undoubtedly, these possibilities, again still very much speculative at this stage, warrant further exploration for the potential of directional sensitivity markers to improve our understanding of CBF regulation post-stroke.

### Limitations of the study

Breathing 5% CO_2_ in air could have led to changes in MCA diameter, thus affecting the relationship between MCAv and absolute CBF.^51,52^ This could have led to an underestimation of MCAv during hypercapnia, although in our case, EtCO_2_ did not reach that reported in MRI studies.^51,52^ Dilation of the MCA during hypercapnia was less likely to distort estimates of ARI_ORIG_, ARI_+D_ or ARI_-D_, because these parameters are not dependent on amplitude of MCAv, only being affected by the temporal pattern of SRMCAv .

When examining the effects of sex on directional sensitivity, we have not taken into account the phase of the menstrual cycle and this limitation needs to be overcome in future studies. The extent to which the lack of control for the menstrual cycle could have affected our results is difficult to anticipate, but there are reasons to believe that this would have been minimal. Firstly, we have enrolled a relatively large number of female subjects (n = 58), which would tend to average out any differences due to participants at different phases of their menstrual cycle and Figure 4 does not suggest a sudden drop in ARI_+D_ for women aged above 50 years. Secondly, previous studies failed to demonstrate an association between the menstrual cycle phase and dCA^53,54^ or VMR_CO2_.^55^ Nevertheless, it is important that future studies enroll a larger number of female participants to increase the statistical power to assess the influence of menstrual cycle phase on dCA, with particular focus on the phenomenon of directional sensitivity.

Of note, we estimated the SRMCAv using segments of data with different duration, that is 5 min for poikilocapnia and 90 s for hypercapnia. One advantage of the ARMA approach, as compared to classical transfer function analysis for example, is that it is less sensitive to the effects of record length.^31^ For this reason, it is unlikely that differences in recording duration could explain the lack of difference in directional sensitivity strength, between poikilocapnia and hypercapnia (Table 2), but future studies using similar recording lengths for the two conditions would be of interest.

## Data Availability

The data that support the findings of this study ae available from the corresponding author upon reasonable request.
